# *catena*-Poly[[[tetra­aqua­zinc(II)]-μ-4,4′-bipyridine-κ^2^ *N*:*N*′] naphthalene-1,5-disulfonate]

**DOI:** 10.1107/S1600536809032127

**Published:** 2009-08-19

**Authors:** Jing Lin, Wen-Lian Cai

**Affiliations:** a Department of Pharmacy, Xiamen University, Xiamen, Fujian 363105, People’s Republic of China; b Department of Chemistry and Environmental Science, Zhangzhou Normal University, Zhangzhou, Fujian 363000, People’s Republic of China

## Abstract

In the title complex, {[Zn(C_10_H_8_N_2_)(H_2_O)_4_](C_10_H_6_O_6_S_2_)}_*n*_, the [Zn(4,4′-bipy)(H_2_O)_4_]^2+^ (4,4′-bipy is 4,4′-bipyridine) cations are linked into linear chains along [001] by the 4,4′-bipy ligands. The Zn^II^ ion exhibits a slightly distorted octa­hedral coordination geometry in which the four water mol­ecules  are in the equatorial positions. The anions are hydrogen bonded to the polycationic chains by O—H⋯O hydrogen bonds, forming a three-dimensional network. The Zn^II^ ion, 4,4′-bipy ligand and anion lie on special positions of 2/*m* site symmetry.

## Related literature

For the design, preparation and applications of metal-organic hybrid materials, see: Batten & Robson (1998 ▶); Hagrman *et al.* (1999 ▶); Cui *et al.* (2003 ▶). For the structural and photoluminescent properties of *d* ^10^ metal (such as Zn) complexes, see: Li *et al.* (2003 ▶); Sattarzadeh *et al.* (2009 ▶). 4,4′-Bipyridine can be used to assembly many transition metal coordination polymers through covalent or hydrogen bonds, see: Yaghi & Li (1995 ▶, 1996 ▶).
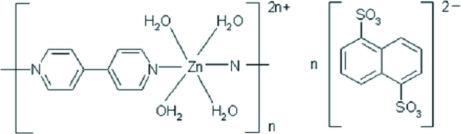


## Experimental

### 

#### Crystal data

[Zn(C_10_H_8_N_2_)(H_2_O)_4_](C_10_H_6_O_6_S_2_)
*M* *_r_* = 579.89
Monoclinic, 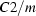

*a* = 14.584 (3) Å
*b* = 7.3948 (15) Å
*c* = 11.380 (2) Å
β = 108.38 (3)°
*V* = 1164.7 (4) Å^3^
*Z* = 2
Mo *K*α radiation
μ = 1.29 mm^−1^
*T* = 293 K
0.38 × 0.29 × 0.19 mm

#### Data collection

Siemens SMART CCD area-detector diffractometer
Absorption correction: multi-scan (*SADABS*; Sheldrick, 1996 ▶) *T*_min_ = 0.658, *T*_max_ = 0.794
5711 measured reflections
1421 independent reflections
1302 reflections with *I* > 2σ(*I*)
*R*_int_ = 0.033

#### Refinement

*R*[*F* ^2^ > 2σ(*F* ^2^)] = 0.034
*wR*(*F* ^2^) = 0.107
*S* = 1.03
1421 reflections
107 parameters
15 restraints
H atoms treated by a mixture of independent and constrained refinement
Δρ_max_ = 0.46 e Å^−3^
Δρ_min_ = −0.52 e Å^−3^

### 

Data collection: *SMART* (Siemens, 1994 ▶); cell refinement: *SAINT* (Siemens, 1994 ▶); data reduction: *SAINT* program(s) used to refine structure: *SHELXL97* (Sheldrick, 2008 ▶); molecular graphics: *SHELXTL* (Sheldrick, 2008 ▶); software used to prepare material for publication: *SHELXTL*.

## Supplementary Material

Crystal structure: contains datablocks I, global. DOI: 10.1107/S1600536809032127/ng2626sup1.cif

Structure factors: contains datablocks I. DOI: 10.1107/S1600536809032127/ng2626Isup2.hkl

Additional supplementary materials:  crystallographic information; 3D view; checkCIF report

## Figures and Tables

**Table 1:** Hydrogen-bond geometry (Å, °)

*D*—H⋯*A*	*D*—H	H⋯*A*	*D*⋯*A*	*D*—H⋯*A*
O1w—H1wa⋯O3	0.85 (2)	1.92 (2)	2.763 (3)	175 (3)
O1w—H1wb⋯O2^i^	0.83 (2)	1.95 (2)	2.768 (3)	166 (3)

Symmetry code: (i) 
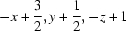
.

**Fig. 1.**
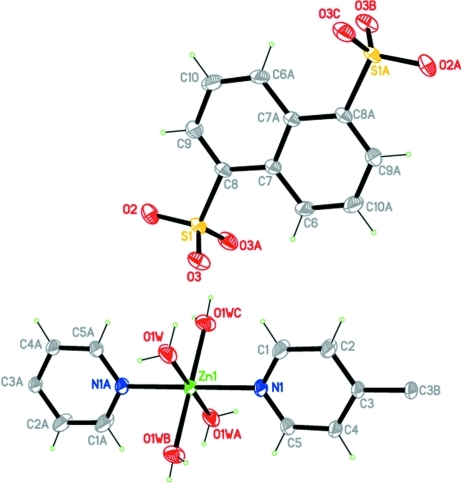
View of the structure of compound (I), showing the atom-labelling scheme. Displacement ellipsoids are drawn at the 35% probability level; H atoms have been omitted for clarity.

**Fig. 2.**
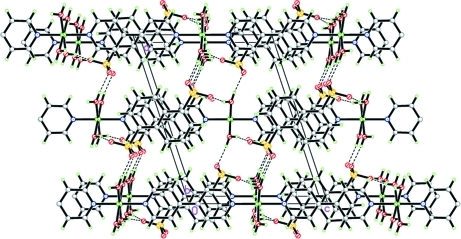
View of the 3D hydrogen-bonded network in the packing of the title compound. The packing is viewed along the b axis; O-H···O interactions are shown as dashed lines.

## supplementary crystallographic information

### Comment

The design and preparation of metal-organic hybrid materials have been studied widely during the past decade owing to their intriguing structures and potential practical applications. (Hagrman *et al.*, 1999; Batten & Robson, 1998; Cui *et al.*, 2003). It has been demonstrated that many d^10^ metal(such as Zn) complexes present intriguing structural and photoluminescent properties (Li *et al.*,2003; Sattarzadeh *et al.*, 2009). On the other hand, bridged ligand,4,4'-bipyridine, can be efficiently used to assembly many interesting transition metal coordination polymers through covalent or hydrogen bonds (Yaghi & Li, 1995, 1996). In this work, we use 4,4'-bpy, 1, 5-naphthalenedisulphonic acid(NDS) and Zn(OAc)_2_ to synthesize a novel 1-D chain polymer through hydrothermal synthesis.

Complex (I) consists of one-dimensional chains formed by 4,4'- bipy ligands through connecting Zn atoms,uncoordinated NDS^2-^ anions, as shown in Fig. 1. The [Zn(4,4'-bipy)(H_2_O)_4_]^2+^ cation is located on a twofold rotation axis that passes through atoms Zn1, N1 and C3. In the cation, the Zn1 atom exhibits slightly distorted octahedral coordination geometry, completed by four O atoms from four water molecules in the equatorial positions and two *N* donors from two 4,4'-bipy ligands in the apical positions. These Zn–O and Zn–N distances are 2.127 (2) and 2.131 (2) Å, respectively. The 4,4'-bipy ligand acts as bis-monodentate linkers and bridge adjacent Zn centers with the Zn···Zn separation of 11.380 (2)Å into an infinite one-dimensional chain.

In complex (I), the NDS^2-^ anions are not involved in coordination but hydrogen-bondedto the [Zn(4,4'-bipy)(H_2_O)_4_]^2+^ cations through the sulfonate oxygen atoms and the coordinated oxygen atoms, forming a three-dimensional hydrogen-bonding network, as shown in Fig. 2.

### Experimental

The hydrothermal reaction of Zn(OAc)_2_.2H_2_O (0.5707 g,2.6 mmol), 1,5-Naphthalenedisulphonic acid(0.5405 g, 1.5 mmol), 4,4'-bipyridine(0.4681 g, 3.0 mmol) and water (15 ml) was carried out at 443 K for 3 d. After cooling to room temperature at 5 K h^-1^, the colorless block crystalline complex, (I), was isolated in 49% yield (based on Zn).

### Refinement

H atoms attached to C atoms were positioned geometrically and refined using a riding model, with C–H = 0.93 Å, and *U*_iso_(H)= 1.2*U*_eq_(C); Water H atoms were located in a difference map and refined with O–H and H···H distance restraints of 0.85 (2) and 1.39 (2) Å, respectively, and with *U*_iso_(H)= 1.5*U*_eq_(O). During the refinement, the displacement parameters of the C1 and C2 atoms were restrained to an approximately isotropic behaviour.

### Crystal data

**Table:**

[Zn(C_10_H_8_N_2_)(H_2_O)_4_](C_10_H_6_O_6_S_2_)	*F*(000) = 596
*M**_r_* = 579.89	*D*_x_ = 1.654 Mg m^−^^3^
Monoclinic, *C*2/*m*	Mo *K*α radiation, λ = 0.71073 Å
Hall symbol: -C 2y	Cell parameters from 5711 reflections
*a* = 14.584 (3) Å	θ = 3.1–27.4°
*b* = 7.3948 (15) Å	µ = 1.29 mm^−^^1^
*c* = 11.380 (2) Å	*T* = 293 K
β = 108.38 (3)°	Block, colorless
*V* = 1164.7 (4) Å^3^	0.38 × 0.29 × 0.19 mm
*Z* = 2	

### Data collection

**Table:**

Siemens SMART CCD area-detector diffractometer	1421 independent reflections
Radiation source: fine-focus sealed tube	1302 reflections with *I* > 2σ(*I*)
graphite	*R*_int_ = 0.033
ω scans	θ_max_ = 27.4°, θ_min_ = 3.1°
Absorption correction: multi-scan (*SADABS*; Sheldrick, 1996)	*h* = −18→18
*T*_min_ = 0.658, *T*_max_ = 0.794	*k* = −9→9
5711 measured reflections	*l* = −13→14

### Refinement

**Table:**

Refinement on *F*^2^	Primary atom site location: structure-invariant direct methods
Least-squares matrix: full	Secondary atom site location: difference Fourier map
*R*[*F*^2^ > 2σ(*F*^2^)] = 0.034	Hydrogen site location: inferred from neighbouring sites
*wR*(*F*^2^) = 0.107	H atoms treated by a mixture of independent and constrained refinement
*S* = 1.03	*w* = 1/[σ^2^(*F*_o_^2^) + (0.0663*P*)^2^ + 1.4457*P*] where *P* = (*F*_o_^2^ + 2*F*_c_^2^)/3
1421 reflections	(Δ/σ)_max_ = 0.001
107 parameters	Δρ_max_ = 0.46 e Å^−^^3^
15 restraints	Δρ_min_ = −0.52 e Å^−^^3^

### Special details

**Table:**

Geometry. All e.s.d.'s (except the e.s.d. in the dihedral angle between two l.s. planes) are estimated using the full covariance matrix. The cell e.s.d.'s are taken into account individually in the estimation of e.s.d.'s in distances, angles and torsion angles; correlations between e.s.d.'s in cell parameters are only used when they are defined by crystal symmetry. An approximate (isotropic) treatment of cell e.s.d.'s is used for estimating e.s.d.'s involving l.s. planes.
Refinement. Refinement of *F*^2^ against ALL reflections. The weighted *R*-factor *wR* and goodness of fit *S* are based on *F*^2^, conventional *R*-factors *R* are based on *F*, with *F* set to zero for negative *F*^2^. The threshold expression of *F*^2^ > σ(*F*^2^) is used only for calculating *R*-factors(gt) *etc*. and is not relevant to the choice of reflections for refinement. *R*-factors based on *F*^2^ are statistically about twice as large as those based on *F*, and *R*- factors based on ALL data will be even larger.

### Fractional atomic coordinates and isotropic or equivalent isotropic displacement parameters (Å^2^)

**Table:**

	*x*	*y*	*z*	*U*_iso_*/*U*_eq_	
Zn1	0.5000	0.5000	0.5000	0.0328 (2)	
S1	0.84039 (6)	0.5000	0.70300 (8)	0.0323 (3)	
O1W	0.60582 (14)	0.7084 (3)	0.54834 (19)	0.0443 (5)	
H1WA	0.6631 (16)	0.698 (5)	0.596 (2)	0.053*	
H1WB	0.605 (2)	0.790 (4)	0.498 (3)	0.053*	
O2	0.8672 (2)	0.5000	0.5896 (2)	0.0451 (7)	
O3	0.78840 (14)	0.6629 (3)	0.71577 (18)	0.0425 (5)	
C1	0.5820 (3)	0.5000	0.7822 (4)	0.0669 (15)	
H1A	0.6403	0.5000	0.7651	0.080*	
C2	0.5848 (3)	0.5000	0.9042 (4)	0.0647 (14)	
H2A	0.6440	0.5000	0.9667	0.078*	
C3	0.4997 (3)	0.5000	0.9346 (3)	0.0324 (8)	
C4	0.4162 (3)	0.5000	0.8359 (3)	0.0364 (8)	
H4A	0.3568	0.5000	0.8501	0.044*	
C5	0.4189 (3)	0.5000	0.7155 (3)	0.0337 (8)	
H5A	0.3607	0.5000	0.6511	0.040*	
C6	0.8693 (3)	0.5000	0.9887 (4)	0.0580 (14)	
H6A	0.8090	0.5000	0.9283	0.070*	
C7	0.9543 (2)	0.5000	0.9528 (3)	0.0329 (8)	
C8	0.9520 (3)	0.5000	0.8266 (3)	0.0338 (8)	
C9	1.0349 (3)	0.5000	0.7957 (4)	0.0549 (13)	
H9A	1.0322	0.5000	0.7130	0.066*	
C10	1.1247 (3)	0.5000	0.8898 (5)	0.080 (2)	
H10A	1.1812	0.5000	0.8684	0.096*	
N1	0.5005 (2)	0.5000	0.6874 (3)	0.0371 (7)	

### Atomic displacement parameters (Å^2^)

**Table:**

	*U*^11^	*U*^22^	*U*^33^	*U*^12^	*U*^13^	*U*^23^
Zn1	0.0270 (3)	0.0560 (4)	0.0140 (3)	0.000	0.0046 (2)	0.000
S1	0.0278 (5)	0.0414 (5)	0.0202 (4)	0.000	−0.0030 (3)	0.000
O1W	0.0347 (10)	0.0589 (13)	0.0301 (10)	−0.0062 (9)	−0.0031 (8)	0.0086 (9)
O2	0.0468 (16)	0.0607 (18)	0.0213 (13)	0.000	0.0014 (12)	0.000
O3	0.0360 (10)	0.0440 (11)	0.0372 (10)	0.0048 (8)	−0.0033 (8)	−0.0028 (8)
C1	0.036 (2)	0.137 (4)	0.030 (2)	0.000	0.0134 (18)	0.000
C2	0.032 (2)	0.135 (4)	0.027 (2)	0.000	0.0088 (17)	0.000
C3	0.0311 (18)	0.048 (2)	0.0190 (17)	0.000	0.0089 (14)	0.000
C4	0.0294 (17)	0.058 (2)	0.0230 (17)	0.000	0.0106 (14)	0.000
C5	0.0302 (17)	0.050 (2)	0.0186 (16)	0.000	0.0044 (13)	0.000
C6	0.0194 (17)	0.120 (5)	0.029 (2)	0.000	−0.0003 (15)	0.000
C7	0.0241 (17)	0.046 (2)	0.0247 (17)	0.000	0.0027 (15)	0.000
C8	0.0244 (16)	0.049 (2)	0.0224 (16)	0.000	−0.0008 (13)	0.000
C9	0.034 (2)	0.106 (4)	0.0219 (18)	0.000	0.0048 (16)	0.000
C10	0.025 (2)	0.178 (7)	0.036 (3)	0.000	0.0093 (19)	0.000
N1	0.0307 (15)	0.065 (2)	0.0157 (13)	0.000	0.0072 (12)	0.000

### Geometric parameters (Å, °)

**Table:**

Zn1—O1W^i^	2.127 (2)	C3—C4	1.372 (5)
Zn1—O1W	2.127 (2)	C3—C3^iv^	1.485 (6)
Zn1—O1W^ii^	2.127 (2)	C4—C5	1.383 (5)
Zn1—O1W^iii^	2.127 (2)	C4—H4A	0.9300
Zn1—N1^i^	2.131 (3)	C5—N1	1.326 (5)
Zn1—N1	2.131 (3)	C5—H5A	0.9300
S1—O3^ii^	1.455 (2)	C6—C10^v^	1.358 (6)
S1—O3	1.455 (2)	C6—C7	1.421 (5)
S1—O2	1.461 (3)	C6—H6A	0.9300
S1—C8	1.784 (4)	C7—C7^v^	1.424 (7)
O1W—H1WA	0.846 (19)	C7—C8	1.426 (5)
O1W—H1WB	0.83 (2)	C8—C9	1.362 (6)
C1—N1	1.330 (6)	C9—C10	1.406 (6)
C1—C2	1.376 (6)	C9—H9A	0.9300
C1—H1A	0.9300	C10—C6^v^	1.358 (6)
C2—C3	1.390 (6)	C10—H10A	0.9300
C2—H2A	0.9300		
			
O1W^i^—Zn1—O1W	180.00 (9)	C3—C2—H2A	119.8
O1W^i^—Zn1—O1W^ii^	87.13 (12)	C4—C3—C2	115.3 (3)
O1W—Zn1—O1W^ii^	92.87 (12)	C4—C3—C3^iv^	123.0 (4)
O1W^i^—Zn1—O1W^iii^	92.87 (12)	C2—C3—C3^iv^	121.7 (4)
O1W—Zn1—O1W^iii^	87.13 (12)	C3—C4—C5	121.1 (3)
O1W^ii^—Zn1—O1W^iii^	180.000 (1)	C3—C4—H4A	119.4
O1W^i^—Zn1—N1^i^	88.18 (8)	C5—C4—H4A	119.4
O1W—Zn1—N1^i^	91.82 (8)	N1—C5—C4	123.1 (3)
O1W^ii^—Zn1—N1^i^	91.82 (8)	N1—C5—H5A	118.4
O1W^iii^—Zn1—N1^i^	88.18 (8)	C4—C5—H5A	118.4
O1W^i^—Zn1—N1	91.82 (8)	C10^v^—C6—C7	120.7 (4)
O1W—Zn1—N1	88.18 (8)	C10^v^—C6—H6A	119.7
O1W^ii^—Zn1—N1	88.18 (8)	C7—C6—H6A	119.7
O1W^iii^—Zn1—N1	91.82 (8)	C6—C7—C7^v^	118.6 (4)
N1^i^—Zn1—N1	180.0	C6—C7—C8	122.9 (3)
O3^ii^—S1—O3	111.78 (18)	C7^v^—C7—C8	118.5 (4)
O3^ii^—S1—O2	112.40 (11)	C9—C8—C7	121.3 (3)
O3—S1—O2	112.40 (11)	C9—C8—S1	117.4 (3)
O3^ii^—S1—C8	107.20 (10)	C7—C8—S1	121.3 (3)
O3—S1—C8	107.20 (10)	C8—C9—C10	119.6 (4)
O2—S1—C8	105.36 (17)	C8—C9—H9A	120.2
Zn1—O1W—H1WA	126 (3)	C10—C9—H9A	120.2
Zn1—O1W—H1WB	120 (2)	C6^v^—C10—C9	121.3 (4)
H1WA—O1W—H1WB	108 (3)	C6^v^—C10—H10A	119.3
N1—C1—C2	123.5 (4)	C9—C10—H10A	119.3
N1—C1—H1A	118.2	C5—N1—C1	116.5 (3)
C2—C1—H1A	118.2	C5—N1—Zn1	121.4 (2)
C1—C2—C3	120.5 (4)	C1—N1—Zn1	122.1 (3)
C1—C2—H2A	119.8		
			
N1—C1—C2—C3	0.000 (2)	C7—C8—C9—C10	0.000 (2)
C1—C2—C3—C4	0.000 (2)	S1—C8—C9—C10	180.000 (2)
C1—C2—C3—C3^iv^	180.000 (2)	C8—C9—C10—C6^v^	0.000 (2)
C2—C3—C4—C5	0.000 (1)	C4—C5—N1—C1	0.000 (2)
C3^iv^—C3—C4—C5	180.000 (1)	C4—C5—N1—Zn1	180.000 (1)
C3—C4—C5—N1	0.000 (2)	C2—C1—N1—C5	0.000 (1)
C10^v^—C6—C7—C7^v^	0.000 (2)	C2—C1—N1—Zn1	180.000 (1)
C10^v^—C6—C7—C8	180.000 (2)	O1W^i^—Zn1—N1—C5	−46.47 (6)
C6—C7—C8—C9	180.000 (2)	O1W—Zn1—N1—C5	133.53 (6)
C7^v^—C7—C8—C9	0.000 (2)	O1W^ii^—Zn1—N1—C5	−133.53 (6)
C6—C7—C8—S1	0.000 (1)	O1W^iii^—Zn1—N1—C5	46.47 (6)
C7^v^—C7—C8—S1	180.000 (1)	N1^i^—Zn1—N1—C5	0(100)
O3^ii^—S1—C8—C9	119.92 (10)	O1W^i^—Zn1—N1—C1	133.53 (6)
O3—S1—C8—C9	−119.92 (10)	O1W—Zn1—N1—C1	−46.47 (6)
O2—S1—C8—C9	0.0	O1W^ii^—Zn1—N1—C1	46.47 (6)
O3^ii^—S1—C8—C7	−60.08 (10)	O1W^iii^—Zn1—N1—C1	−133.53 (6)
O3—S1—C8—C7	60.08 (10)	N1^i^—Zn1—N1—C1	180 (100)
O2—S1—C8—C7	180.000 (1)		

Symmetry codes: (i) −*x*+1, −*y*+1, −*z*+1; (ii) *x*, −*y*+1, *z*; (iii) −*x*+1, *y*, −*z*+1; (iv) −*x*+1, −*y*+1, −*z*+2; (v) −*x*+2, −*y*+1, −*z*+2.

### Hydrogen-bond geometry (Å, °)

**Table:**

*D*—H···*A*	*D*—H	H···*A*	*D*···*A*	*D*—H···*A*
O1w—H1wa···O3	0.85 (2)	1.92 (2)	2.763 (3)	175 (3)
O1w—H1wb···O2^vi^	0.83 (2)	1.95 (2)	2.768 (3)	166 (3)

Symmetry codes: (vi) −*x*+3/2, *y*+1/2, −*z*+1.
